# Cultural lenses of the utility of the ICD-11-PD model: Integrating the Peruvian context

**DOI:** 10.3389/fpsyt.2022.1016471

**Published:** 2022-10-05

**Authors:** Luis Hualparuca-Olivera

**Affiliations:** Departamento de Humanidades, Universidad Continental, Huancayo, Peru

**Keywords:** personality disorder, dimensional model, ICD-11, clinical practice, Peruvian culture

## Introduction

Personality disorder (PD) is a common condition in Western countries. The new ICD-11 dimensional model of PD based mainly on the severity of self and interpersonal dysfunction appears to be a relatively viable solution in low- and middle-income countries (LMICs) to facilitate clinical practice. Culture is known to affect the development and course of PD, as well as shape PD evaluation and diagnosis, treatment, and research. In this article, I explain the challenges posed by Peruvian cultural characteristics in clinical practice and the limited usefulness of the ICD-11 PD model, due to the sociopolitical interference of health authorities, corruption, and the lack of qualification of professionals. In addition, I highlight the interethnic characteristics that complicate effective communication of symptoms between the patient and the diagnostician, especially in the rural and high Andean areas of Peru.

## Peruvian culture as a contextualizing agent of personality

Culture is a system of identity construction processes that is socially mediated by the negotiation of interpersonal relationships and participation in local and global communities and institutions (1). This system plays a significant role in the construction of self-concept, self-image, interpersonal schemes, and social norms (2). Therefore, cultural contextualization is crucial in the demarcation between normal personality and personality disorder (3), or even in the conceptualization of a personality that is more than adapted or positive (4–6). Certainly, the cultural view of development, the current conceptualization of personality and the diagnosis of PD must always be complemented by biopsychosocial frameworks (7). So, when evaluating culture in PD, four components must be taken into account: subjective perceptions of the group, individual situations, social structures, and the regional geographic setting (2).

Peru is an intercultural and multicultural country with various ethnic groups that share the same norms, environments, and experiences. This country has 55 indigenous peoples, three natural geographic regions (coast, mountains and jungle), three official languages (Spanish, Quechua and Aymara) and 47 original languages (8). Peruvians are typically monolingual in urban areas and multilingual in rural areas. Through the use of their languages, the Peruvian people preserve and transmit their affections, traditions, worldviews, values and knowledge to the next generations and to the world (9). This is why languages are an essential part of the cultural identity of people and shape the personality of individuals. Spanish colonization through unequal treatment, times of poverty, hyperinflation, internal armed conflict, illicit drug trafficking, social conflicts, and other events, have permeated the mental health and identity of the Peruvian aborigine (10). The indigenous people of this country used to live in rural areas, but due to a lack of opportunities they had to migrate to urban areas and to Peruvian capital, where they were often victims of discrimination due to their skin color, language and customs. In this sense, the effects of acculturation by themselves should not be confused as a symptom of PD, such, impulsiveness, instability, affective lability, explosive/aggressive behavior or dissociative symptoms of the borderline pattern (11, 12). However, it cannot be denied that the search for better opportunities has influenced the generation of conflicts and has collaborated with the development or course of PD.

Prevalence investigations in Peru have left PD relegated; however, studies of other mental disorders can serve as a link to identify the presence of PD; given its comorbidity according to the Hierarchical Taxonomy of Psychopathology, HiTOP (13). For example, according to a national study (14), it is evident that in Peruvian women there is a higher prevalence of internalizing symptoms, while in men externalizing manifestations prevail. Likewise, in the highlands of Peru there is a higher prevalence of internalizing disorders (generalized anxiety and depression) and externalizing disorders (alcohol consumption and psychopathic tendencies), while in the jungle mental disorders are infrequent. With the biopsychosocial framework and considering some limitations that characterize this study, it can be inferred that people from the Peruvian highlands have a greater number of vulnerabilities. Likewise, unequal upbringing (often sexist) is added to differentiated sexual characteristics, which generates dissocial tendencies and disinhibition in men; and causes tendencies of negative affectivity in women. Apparently this situation is not very common on the Peruvian coast or in the jungle, since the legacy of violence in the armed conflict has not affected them as much as it has in the Peruvian highlands. It should be noted that these inferences must be taken with caution in order to adequately differentiate PD from mental disorders; since the latter, although they can manifest in a similar way in a multiplicity of circumstances, generally do not present additional characteristics that generate dysfunction [i.e., trait domain specifiers, (12)].

## Role of culture in ICD-11 personality disorders

In ICD-11, PD is defined mainly by self or interpersonal dysfunction, which is measured by mild, moderate or severe thresholds; as well as by assigning trait domain specifiers, and borderline pattern. The main requirement of PD severity can be evaluated using the four components of Choudhary and Gupta (2) described above. In addition, some authors (3, 15) mentioned the roles of culture in PD that can be easily applied to the ICD-11 guidelines: as an interpretive/explanatory tool, pathogenic/pathoplastic agent, diagnostic/investigative factor and element psychotherapeutic. Culture influences the interpretation of PD, since the diagnosis can only be assigned if the dysfunctional behavior is far from the cultural characteristics of a society or biasedly attributed according to certain idiosyncratic conceptions (15). Culture also configures a pathogenic agent, since (traditional) Eastern societies tend to be more collectivist, causing dependency, separation anxiety or other patterns of negative affectivity in a maladaptive extreme (15, 16). Likewise, in Western societies which are often individualistic due to modernity and competition, egocentric, and other dissocial characteristics are commonly observed (15, 16).

Culture is also a clinical and research tool for PD since it provides guidelines for the identification of acculturation, linguistic abilities and preferences, meaning of stressful experiences, explanatory models of the disease, perceived levels of adaptive functioning, attention, search patterns, expectations for the outcome of the disease, quality of life and the relationship with the diagnostician/physician (7, 15). Similarly, cultural factors are relevant to psychotherapy (the language to be used, the cultural background of the therapist and the patient, personality styles, identity, attitudes and beliefs, and relationships with authority figures), as well as for pharmacokinetic and pharmacodynamic processes, prescription guidelines, treatment compliance and outcome measurement (15). In addition to the roles mentioned, Table 1 provides a general description of the cultural aspects of PD according to ICD-11. Since the ICD-11 PD model, only one study has been carried out on male inmates in a prison in the Peruvian highlands, mainly finding differences according to the age of the participants and the crime committed, since the general psychopathology included in PD is more common in those under 35 years of age and in crimes against property (5). More studies with populations from diverse cultural contexts are needed to gain a better understanding of the subject.

**Table 1:** Culture-related features.

• Assessment of personality across cultures is challenging, requiring knowledge of normative personality function for the sociocultural context, variations in cultural concepts of the self, and evidence for consistent traits and behaviors across time and multiple social contexts. • Culture shapes modes of self-construal, social presentation, and levels of insight about behaviors that are related to personality development, including what are considered normal and abnormal personality states in a given setting. For example, children reared in collectivist societies may develop attachment styles and traits that are viewed as dependent or avoidant related to the norms of more individualistic cultures. In turn, traits of self-involvement that are accepted or positively valued in individualistic cultures may be considered narcissistic in collectivist cultures. • Diagnosis of Personality Disorder must take into account the person's cultural background. Collateral information may be needed to assess whether certain disruptive self-states and behaviors are considered culturally uncharacteristic and therefore consistent with Personality Disorder in a given culture. In general, a diagnosis of Personality Disorder should be assigned only when the symptoms exceed thresholds that are normative for the socio-cultural context. • Among ethnic minority, immigrant, and refugee communities, responses to discrimination, social exclusion, and acculturative stress may be confused with Personality Disorder. For example, suspiciousness or mistrust may be common in situations of endemic racism and discrimination. • Socio-cultural contexts of exclusion affecting marginal social groups can evoke repeated attempts at self-affirmation or acceptance by others that are based on ambiguous or troubled relationships with authority figures and limited adaptability. These reactions may be confounded with manifestations of Borderline pattern, such as impulsivity, instability, affective lability, explosive/aggressive behavior or dissociative symptoms. However, a diagnosis should be assigned only when the symptoms exceed thresholds that are normative for the socio-cultural context.

Obtained from https://icd.who.int/browse11/l-m/en#/http%3a%2f%2fid.who.int%2ficd%2fentity%2f941859884 (17).

## Discussion

Considering the component of social structures (2), the evaluation and diagnosis of PD with the ICD-11 model will probably not have a major impact on Peruvian clinical practice from the state sector that covers health insurance for the majority of the Peruvian population. This is because, as in many LMICs, the priority of diagnosis through regulation and investment is given to certain disorders (e.g., anxiety, depression, psychosis, and substance use) that are often remitted through brief interventions (e.g., 10 sessions). There are even quantifiable goals established by period for primary care; in specialized care the diagnosis is often an arbitrary sociopolitical consensus based on interests in the production of services, and on the stigma on the part of professionals who do not want to diagnose PD because it implies an avoidable complexity for treatment (18). In addition, the PD does not qualify as a valid diagnosis to be subsidized by EsSalud^1^ as a cause of disability. Although the aforementioned is a pessimistic view of the panorama, it is the most likely scenario to arise as has already been evidenced with other issues related to mental health. Even if an effort was made to adequately train public health servants, it would be unsuccessful. This is due to the political interference of leaders of health entities, arbitrary hiring of unqualified professionals, among others; It prevents an adequate and pertinent differential diagnosis of PD from being made. Despite this, the new PD model can be beneficial to private clinical practice, which is not usually greatly affected by these problems.

Another factor that is often not considered owing to the same weaknesses already described is the subjective perceptions of the group. Health professionals in Peru are constantly rotating, and many work with populations with little or no knowledge of customs or their own language. Consequently, an inadequate diagnosis can be generated when evaluating rural residents of Andean and jungle mining areas, who are in constant social conflict with companies in the industry to defend their natural resources and may express mistrust, anger and emotional instability; which can be confused with borderline pattern traits. Likewise, the employees of these mining companies often show patterns of isolation due to institutionalized working hours which can be confused with traits of detachment from a diagnostician's perception that is alien to these customs (19). Quechua-speaking victims of armed conflict and their families, who live in the Andean zone at more than 4,000 meters above sea level or far from state protection, often present patterns of depression, separation anxiety in women, and alcohol consumption in men and aggressiveness (14); which can be confused with patterns of negative affectivity and disinhibition, respectively. It is likely that the therapeutic bond is also affected by these differences between the cultural conceptions of the therapist and patient.

It is common to observe in consultation the desertion of patients to attend their care, including the inconvenience generated when trying to make home visits. In this sense, only moderate or severe personality disorder (with prominent ego-syntonic traits) that implies considerable anguish can be seen in voluntary care in Peruvian health centers and community mental health centers, while mild PD will probably be considered normal (16). The moderate and severe PD (with prominent ego-dystonic characteristics) suffered by men will be evidenced mainly when they are involved in violent crimes; while mild PD is often camouflaged in the form of violence that is not reported to the authorities and is arranged by the patient's relatives given the chauvinistic characteristics of Peruvian society.

Although in clinical evaluation the diagnosis of PD is avoided, in Peruvian legal environments there seems to be an affinity of the diagnostician for this disorder (18). PD symbolizes a negative diagnosis that is often used to disqualify a person; and facilitate the legal process for criminals. Imprisonment is a mechanism for infringing punishment rather than correcting sentenced and processed Peruvians. Many Peruvian prisons do not even have psychologists or health professionals, since they are added to corruption, lack of investment and lack of training in the mental health of legal actors, making it difficult to properly diagnose PD. It is likely that the problem of diagnosis and adequate treatment of PD in the case of Peruvian convicts continues to persist due to a generalized failure of the Peruvian state system and the very values transmitted among Peruvians from the early stages of their development.

PD research in Peru is scarce and mostly developed with inadequate methodology (19). In general, mental health is not an essential issue for governments of the day and research itself is an area that has recently been promoted by the agencies in charge. Although the general panorama is unfavorable for clinical practice and research on PD, it is also important to highlight the efforts of some professionals with a vocation for service and emerging researchers to develop the bases of knowledge and praxis of PD that will be reflected in the long term. In fact, it is urgent that these matters be improved since, with the modernization, centralization and alienation of the Peruvian by the American individualistic model, Peruvian urban societies become a “breeding ground” for the expansion of dissocial tendencies, and possible patterns of detachment in adolescents whose parents neglect them for work issues (16).

## Final remarks

In this article, I analyze personality and its pathology in the Peruvian cultural context; as well as the role of culture in the current ICD-11 PD model. Likewise, I highlight the usefulness and implications of this model in the evaluation, treatment, and research of PD in the Peruvian scenario.

## Author contributions

LH-O contributed solely with all the preparation of this paper.

## Funding

The subsidy for the processing payment of this article was covered by the Vice-Rectorate for Research of the Continental University of Peru.

## Conflict of interest

The author declares that the research was conducted in the absence of any commercial or financial relationships that could be construed as a potential conflict of interest.

## Publisher's note

All claims expressed in this article are solely those of the authors and do not necessarily represent those of their affiliated organizations, or those of the publisher, the editors and the reviewers. Any product that may be evaluated in this article, or claim that may be made by its manufacturer, is not guaranteed or endorsed by the publisher.

## Footnotes

^1^A government health insurance entity for the economically active population.
