# Corrigendum: Cultural lenses of the utility of the ICD-11-PD model: Integrating the Peruvian context

**DOI:** 10.3389/fpsyt.2022.1066682

**Published:** 2022-11-04

**Authors:** Luis Hualparuca-Olivera

**Affiliations:** Departamento de Humanidades, Universidad Continental, Huancayo, Peru

**Keywords:** personality disorder, dimensional model, ICD-11, clinical practice, Peruvian culture

In the published article, there was an error regarding the affiliation of “Luis Hualparuca-Olivera.” In the version of this article initially published, Luis Hualparuca-Olivera's affiliation was given as “1. Departamento de Ciencias de la Salud, Universidad Nacional Federico Villarreal EUPG, Lima, Peru, 2. Departamento de Humanidades, Universidad Continental, Huancayo, Peru, 3. Oficina de Desarrollo de Recursos Humanos e Investigación, Dirección Regional de Salud, Huancavelica, Peru.” The correct affiliation is “Departamento de Humanidades, Universidad Continental, Huancayo, Peru.”

In the published article, there was an error in the **Funding** statement. An incorrect funder was previously displayed. The correct **Funding** statement appears below.

## Funding

The subsidy for the processing payment of this article was covered by the Vice-Rectorate for Research of the Continental University of Peru.

The authors apologize for this error and state that this does not change the scientific conclusions of the article in any way. The original article has been updated.

## Publisher's note

All claims expressed in this article are solely those of the authors and do not necessarily represent those of their affiliated organizations, or those of the publisher, the editors and the reviewers. Any product that may be evaluated in this article, or claim that may be made by its manufacturer, is not guaranteed or endorsed by the publisher.

